# From Choirs to Collective Singing Communities: Learning the Art of Care from a Journey Through Lebanon, Mozambique, Benin, and Greece

**DOI:** 10.3390/bs15121615

**Published:** 2025-11-24

**Authors:** Ágata Ricca, Inês Lamela, Paulo Maria Rodrigues

**Affiliations:** INET-MD—Institute of Ethnomusicology: Center for Studies in Music and Dance, Department of Communication and Art, University of Aveiro, 3810-193 Aveiro, Portugal; maria.ines.lamela@ua.pt (I.L.); pmrodrigues@ua.pt (P.M.R.)

**Keywords:** choir, collective singing communities, well-being, ethics of care, cultural diversity

## Abstract

In this paper we discuss a journey through different collective singing experiences held between 2019 and 2023 in Lebanon, Mozambique, Benin, and Greece. It is an autoethnographic work that includes the short-term participant observation of a choir in a context of conflicting neighbourhoods (Lebanon); the long-term experience of teaching choirs in a musical project aiming at social integration (Mozambique); the short-term experience of leading musical activities in a project for orphaned children rescued from voodoo convents (Benin); and the medium-term experience of facilitating music sessions in an organization working with people living in refugee camps (Greece). Ethics of care emerged as an inspiration for reformulating previous European choir tradition practices, adapting it to the reality of each context and supporting the overall experience. Starting from an idea of choir rooted in our European background, this journey transformed our perception of collective singing communities and how we can nurture well-being and build a sense of care within groups. Assuming different types of leadership, adapting the communication to specific circumstances, and being aware of opposite needs for continuity were the main strategies involved in making each experience a unique act of care.

## 1. Introduction

The landscape of choral practice is both broad and diverse. We propose to start this journey with two conceptual coordinates that are commonly accepted by many choir members and conductors trained within the European choral tradition. First, the idea of the choir as a group of people who sing polyphonic repertoire—whether secular or sacred—typically notated in scores, shaped by specific rituals and vocabulary in both rehearsals and performances, and usually led by a conductor who holds the artistic decision-making authority. Second, the idea of choral practice as one of the most inherently inclusive forms of music-making, as it does not require an external musical instrument.

Singing is within almost everyone’s reach and many people engage in choirs because they are spaces of diversity, learning, and friendship, where it is possible to make music with others (de Quadros, 2015). Well-being is often reported as one of the benefits associated with choral singing (Clift & Hancox, 2010; Fernández-Herranz et al., 2021). The choir is not, however, always a safe and comfortable space, either due to the way it is led by the conductor or because of interpersonal problems arising between fellow singers. For Garnett (2005, p. 249), “singing in a choir is a regime of the body”, as the rehearsal process is based on monitoring the singers’ performance, looking for errors or omissions that need to be corrected. As the body is inseparable from the act of singing, and despite choral singing being usually seen as an inclusive activity, a choir exercises authority over the body, not only in relation to physical disposition but also to vocal production. Implicit notions of what an ideal sound and a right voice are, which include a certain timbre, projection, breathing control, diction, and pronunciation, can lead a choir to be a place of exclusion for individuals who do not fit those expectations (or who feel they do not fit in). O’Toole (2005) notes that the various voices that intervene in a choir rarely present themselves in their plurality, to the detriment of a single voice, homogenized by the choir director. The academic training in choir conducting spreads this choral approach and usually ignores the singer’s personal experience.

The initial motivation for this research was to explore and reflect about choral practices that could enhance singers’ well-being and feelings of safety and being heard. The journey allowed us to become acquainted with different environments, eventually leading to questioning one’s own practices and well-established boundaries, to the point where the term “collective singing communities” emerged as a better designation for the initial scope of the research.^1^ This approach resonates with community music principles of hospitality, invitation, shared experience of music making, development of confidence and creativity, acknowledgement of both individual and group ownership, and the respect of the cultural property of the participants and intercultural acceptance and understanding (Higgins, 2012). Recognizing the process (that involves both social and personal growth of the participants) is as important as the artistic result (Henley & Higgins, 2020). These principles can be considered provocative as they shake the well-rooted ideas and practices developed over the years in choral conducting training programmes that place the emphasis in techniques (for conducting, rehearsing, and voice production) and in repertoire. In our case, they created restlessness, opening the urge to investigate, both theoretically and in practice, different approaches to the conventional idea of choir practices.

## 2. Ethics of Care

Although often associated with caring professions (people working directly looking after the needs of others) and women, care must be universal and present in all aspects of life, with practices as broad as possible, whether with acquaintances or strangers, physically near or far. It does not select when or who is cared for and recognizes that we all have the capacity to care, improving our lives by doing so, by being cared for, and by caring together. Ethics of care is currently guiding many different fields of activity, including education and the arts. Authors such as Held (2006), Gilligan (2011), Noddings (2005), Thompson (2022), and Tronto (1993) have nurtured our ideas, and we found the work by The Care Collective (2020) particularly inspiring. According to The Care Collective manifesto, care is a social capacity and activity that involves the nurturing of what is necessary for the flourishing of life (The Care Collective, 2020). It is the “individual and common ability to provide the political, social, material, and emotional conditions that allow the vast majority of people and living creatures on this planet to thrive—along with the planet itself” (The Care Collective, 2020, p. 6). An act of recognizing care for another does not always need to be a grand demonstration and can be something as simple as a smile (Silverman, 2012), a warm greeting, or a small conversation (Hooks, 2018). In this sense, Laughter (2014) introduces the concept of acts of micro-kindness, aiming to improve interactions between people by small actions (as varied as words, body language, or facial expression) that everybody can easily do in their daily lives, in different contexts.

In what concerns music, Bowman (2001) states that an ethics of care is the act of individual investment, not only in a personal action but also in a collective practice. In this way, the ethics of care can bring extra-musical consequences to projects and education, contributing to the development of identity as part of a collective. Silverman (2012) states that by facilitating caring relationships, the possibility of living transformative musical experiences is enhanced, creating positive interpersonal relationships and developing a sense of democracy. In a context of musical experience and education, this attitude can create a safe space for individual and collective sharing and is the basis for the development of social justice.

The invitation, the hospitality, and the need to keep the conditions for the well-being of the participants resonate with the principles of community music (McArton & Mantie, 2023; Yi & Kim, 2023). Lately, the ethics of care has also become an important idea for the communication and discussion of projects within this framework. This journey is, hopefully, another contribution to disseminate these concepts as important aspects of making music.

## 3. Methodology

This paper arises from the doctoral research and fieldwork of the first author, a choir conductor, supervised by the second and the third authors. The research project followed rigorous ethical principles approved by the University of Aveiro Ethics Committee. The general aim of the research was to explore ways of creating safe spaces and moments of well-being through the sharing experience of singing together. Working over a range of cultural, social, educational, and musical situations was an important asset of the project and the COVID-19 pandemic greatly influenced the routes of this journey.

The fieldwork was developed between 2019 and 2023 in Lebanon, Mozambique, Benin, and Greece. These different contexts allowed us to learn, by observing and practicing, the idea of dialogue being fundamental in exploring different ways of leading and being led. They alternated with periods of reflection, systematization of information, planning and developing musical/leadership skills. The concept of ethics of care became central in this journey and informed many of the practical decisions, as well as the conceptual framework.

Auto-ethnography^2^ was the methodology used to collect, write, and analyze the data presented in the doctoral project from where this paper arises. Ellis et al. (2011) state that part of auto-ethnographic work is the recording of “epiphanies”, that is, moments in which certain experiences bring fundamental revelations for the journey and help the researcher to have a better understanding of the phenomena under study and improving and adapting practices.

The self-observation and subsequent self-reflection (López-Cano & Opazo, 2014) that guided the fieldwork are in line with action research practices. Auto-ethnography and action research have been related by some authors (Ellis & Bochner, 2000; Hughes & Pennington, 2017; Vang et al., 2023), who consider that auto-ethnography can take the form of an action research due to the usefulness of the cycles of critical self-reflection.

The process of observation, description, and notation (Coutinho, 2020) was the basis for recording brief representations of people, contexts, or events, and creating detailed text reports in logbooks, frequently accompanied by photographs and videos. These data were then analyzed for the doctoral thesis with a broad and flexible set of tools: searching for recurring topics; identifying exceptional occurrences; broadly contextualizing the different experiences in relation to their contexts; and comparing cases (projects/experiences) to understand similarities and differences between them (Chang, 2008). Data analysis was also an introspective process, which led to reflections and discussions that led to the attribution of what Wall (2006) defines as themes and meanings. Regarding the reports on the Nagham Choir’s Facebook page and the responses to the Xiquitsi students’ documentary, data reduction and coding were performed (Coutinho, 2020) to allow these reports to be grouped by themes and to understand which of these themes emerged most frequently: “(…) the researcher looks for patterns of thought or behavior, words, phrases, that is, regularities in the data that justify categorization” (Coutinho, 2020, p. 216). For this paper, the three authors gathered to analyze the auto-ethnographic work performed during the doctoral path of the first author with the goal of communicating the entire experience.

Within the work presented here, there were transitions with undefined boundaries between the roles of choral director, teacher/educator, facilitator, and researcher, according to the context and the skills most needed in each situation. In line with Higgins (2010) suggestion, the presence of the first author as a musician and relational being was of paramount importance in a process that can be defined as “practice based research”, with characteristics such as flexibility for observation, learning and adaptation, based on dialogue, as well as the ability to transfer practical experience (informed by previous experiences and the knowledge transmitted by others) to the creation of theoretical knowledge.

This was a journey through different contexts and realities, where the first author interacted with many people, always as an outsider. The awareness that, despite all the preparation, the knowledge of the local reality is limited and grows over time was always present. All these experiences, in the various roles the researcher played during the journey (conductor/choir director, teacher, and facilitator), were fundamental in questioning the role of group singing in different contexts and with/for different people. It was necessary to always take these specificities into account, using flexible approaches, and the notion that music (or group singing) was not, and is not, in itself, a solution for complex social problems.

A great ethical concern was ever-present throughout the entire process (from choosing the topic, living each experience, and transforming it into writing). This concern was motivated not only by the fact that people’s voices, desires, needs, concerns, and dreams were involved, but also by the need to respect all the cultural differences encountered. In this work, we are aware that we are shaped by our Western European culture and the privileges associated: in one project (Greece), the first researcher travelled freely to work with people who are not allowed to leave their place; in another (Mozambique), we were natives of the country that was colonizing it for centuries. We felt that it is essential to constantly question prejudices (in the sense of an idea that is formulated in advance) and be critical of our practice and privileges, and also discuss these issues together so as not to perpetuate forms of discrimination and imperialism.

## 4. Journey

In this section we present the four distinct field experiences lived in the context of this research: a participant observation in Lebanon (January 2020), in a choir within a context of conflict between two adjoining neighbourhoods; a project that aims at social integration through the collective practice of music in Mozambique, which started with a two-month period in 2019, and that turned later into a choir teacher position for three school years (2020–2022); the creation of a short-term project based on musical practice for orphaned children rescued from voodoo convents in Benin (January 2023); and the facilitation of music sessions in an organization that works with displaced people living in refugee camps in Greece (February to April 2023).

The description that follows reflects the length of the fieldwork and amount of data generated.

### 4.1. Lebanon

The field trip to Lebanon occurred in January 2020 for a period of two weeks and it was a short-term participant observation of a choir’s routines in a context of conflicting neighbourhoods. The main goal was to work within the Fayha Choir, an internationally recognized choir created in 2003 by conductor Barkev Talaskian, with branches in Tripoli, Beirut, and Chouf. Several objectives led to the founding of this choir, as explained by the conductor: to develop and disseminate Arabic music; to share the reality of Lebanon, specifically, and Arab countries, in general, through their music, trying to change the poor image that the world has of the region; to promote collective singing in the Arab countries; and to create an archive of Arabic choral music arrangements. Fayha Choir, with arrangements by Armenian composer Edward Torikian, was one of the first choirs in the Arab world to explore aspects of the polyphonic choral tradition adapted to Arabic music, which is predominantly homophonic, sharing its resources with other choirs. Along with Fayha Choir, conductor Talaskian and his assistants divide their work among other projects with social aims.

One of those projects, observed in the field trip to Lebanon, was the Nagham Choir, a group made up of 30 young residents from two adjoining conflicting neighbourhoods in the city of Tripoli. The roots of the conflict go back to the Lebanese civil war and are mostly related with political questions regarding the neighbour country Syria: in Bab al-Tabbaneh, the residents are Sunnis aligned with the Syrian opposition force; the people living in Jabal Mohsen are Alawites and supported the ex-president Bashar al-Assad and his regime. The choir was created in 2019 with the main objective of building bridges between young people living on opposite sides of this conflict, promoting the differences and richness of the identities of their neighbourhoods, and showing it to external communities.

At the entrance of both neighbourhoods there were tanks and soldiers, and the buildings were marked with bullet holes. A building on the street that separates these two neighbourhoods hosted the Nagham choir rehearsals at the headquarters of the Ruwwad Al-Tanmeya association. A door on each side of the building, facing each neighbourhood, allowed it to be safely accessed by people from both sides.

During the rehearsals observed, it was clear that the conductor made no distinction between individuals from different neighbourhoods and the singers reported that they had become friends over time, regardless of their religion or where they lived. The choir members learned the songs by repetition. There was an enormous effort from the conductor in developing the sound of the choir—its homogeneity, fusion, and intonation. The rehearsal environment was marked by the singers’ great desire to achieve good results, through attention, motivation, and respect for the work developed and for the conductor.

Although the purpose of the visit was to observe and to learn, it was possible to interact musically with the group and to rehearse two songs, the South African song Shosholoza, in a four-part (SATB) arrangement by A. Moll, and the Catalan song Cançon de Bres per Una Princesa Negra, arranged in a canon by A. Rodrigues. This choice of repertoire was as a response to the invitation issued by the choir’s conductor, of challenging daily work, in terms of repertoire and approach.

The Catalan song was learnt without lyrics to avoid the difficulties posed by the long text and short rehearsal time available. The text, however, was not the only difficulty. The melody itself, and its triple metre, also brought some obstacles. As the choir conductor explained, this type of division is not so common in Arabic music and in Nagham’s repertoire. The song Shosholoza, in contrast, was easier to learn: it is short and it has a simple harmony and repetitions. It confirmed previous experiences with other choirs as a piece that gives the group a sense of pleasure in singing together, creating space for experiments with music structure, dynamics, and body movement. The rehearsal environment was different from those attended before, with the initial shyness quickly turned into curiosity for a different way of working led by a different person, culminating in a small presentation. There was also a moment of structured conversation with the members of the choir about the meaning of being a part of the group. Additionally, it was possible to travel to the Bekaa Valley region, at the border with Syria, to work with a refugee status Syrian children’s choir and to follow the rehearsals of the three branches from Fayha Choir. This, too, was a remarkable experience. Besides the musical moments there was time for informal discussions, to become acquainted and enjoy the presence of the community involved in these projects, allowing a better understanding of their culture, lives, and the meaning of singing in a choir.

### 4.2. Mozambique

The fieldwork in Mozambique happened in two circumstances, both connected with the music project Xiquitsi: a first period in July–August 2019, later followed by a second period developed over three academic years (2020–2022). It was a long-term participant experience of teaching choirs in a musical project.

Xiquitsi is a project aiming at social integration and professional training of future musicians through collective music education. It also has the goal of creating the first youth classical orchestra and choir in Mozambique. It is an initiative by Kulungwana—Association for Cultural Development and was founded in 2013 by the Mozambican oboist Eldevina Materula. Xiquitsi does not fit into the definition of a school or formal education, despite some of the terminology used (words like student, teacher, classes, and pedagogic objectives). There is no assessment or grading and the classes do not follow a previously stipulated curriculum. The content is designed by the teachers according to the students’ needs and the artistic goals of the project. Throughout the fieldwork period, Xiquitsi had around 240 students, between the ages of 6 and 25, who had access, mainly, to collective classes (orchestra and choir) but also, in some cases, to individual classes (violin, viola, cello, double bass, clarinet, singing and percussion). There were also classes in ear training, composition, traditional percussion, and lutherie (to repair the string instruments and to build traditional ones, such as mbira nyunga nyunga). Some of the older Xiquitsi students, more experienced and/or more musically advanced, become monitors and teach younger students, always in dialogue with the teachers. This allows them to gain experience, have more responsibility inside the project, receive a salary, and start working professionally as musicians.

The first period in Mozambique was to prepare the Xiquitsi youth choir for the performance of the opera Orpheus in the Underworld, by J. Offenbach. This was a project that had been planned by the institution, who invited the first author to fill in a vacant position in preparing the choir at an ongoing stage. The opera was to be performed by Xiquitsi students, teachers, and some guest soloists. It would be staged, based on adaptations made on the original score, namely the orchestration, re-written for a small instrumental ensemble. The original text in French had been translated into Portuguese and references to food, places, and some terms had been adapted to the Mozambican context and to a local language—Changana. At the time of the arrival—6th of July—the work was still at an early stage, with the first of two performances scheduled for the 9th of August. The plan required the choir to appear frequently on stage, performing from memory, participating in three of the four acts, which correspond to twelve scenes. The choir soloists would have additional work, being present in twenty-four scenes in total. The strategies used in the preparation of the choir and soloists did not differ greatly from those used in other projects within the European choir tradition, but the overall artistic and educational results were unparalleled: what seemed to be an impossible task, considering the little time available, was not, due to the amazing effort of the students, their enthusiasm, willingness to learn, to sing, and to make music together. It was a remarkable and very successful experience, leading to an invitation to return at the beginning of the following academic year.

The second period started in February 2020 and led the first author to be responsible for all Xiquitsi’s choral activities for the following three academic years. In March 2020, however, the rehearsals were forced to stop as a consequence of the pandemic situation and the first author returned to Portugal. During the various periods of confinement that followed, the choir activities continued online, with all the difficulties that this implies, namely the lack of computers or smartphones and difficult access to the internet in many of the students’ homes. The application WhatsApp became the choir’s classroom. Weekly tasks were sent to the students and through the exchange of material, messages, voice messages, calls, and video calls it was possible to work individually with them. To have the feeling of working together and not just individually, the students rehearsed some songs with the aim of creating virtual choirs: each student prepared their video (or audio and a photo of themselves, when their cell phones did not allow video recordings) and they were all edited together, so it looked like it had been recorded with everyone singing in the same room^3^. Throughout 2020 and 2021, this was how the group mostly worked. It was possible, approximately twice a year, for periods of three months, to go back to Mozambique and do some in-person work, resulting in concerts with reduced groups and following all the safety measures.

In 2022, all the activities were held inperson again. As a result of the ongoing research, new practices were introduced in order to include the students in the artistic choices and decisions, giving them more ownership and autonomy and to transform the choir in a less hierarchical structure. Among other activities and efforts, sessions were held where students had the opportunity to conduct each other and show the results of their endeavours in public performances. These presentations dedicated exclusively to choir and vocal music were mostly organized by the students, who chose the repertoire, how they wanted to present themselves and, when applicable, staging and movement.^4^

During this year it was also possible to witness the first steps of the Cantate Project in Nampula and in Cabo Delgado, and to participate in some of its activities. The Cantate Project is another initiative by Kulungwana, in partnership with the Ministry of Culture and Tourism, with the support of the European Union. It aims to provide alternatives to young people from areas in conflict through art and culture. The musical work is based on the experience of Xiquitsi, its management, its teachers, and monitors, with material—repertoire and instruments—and human resources being shared. It started in 2021 in Nampula. Later, in 2022, another branch started in Cabo Delgado, and, in 2024, yet another started in Niassa. The short fieldwork in Nampula had the objective of participating in the auditions to select future participants, while in Cabo Delgado the goal was to help the preparation of the students for the final concert.

### 4.3. Benin

The fieldwork in Benin was developed with two other musicians, following a Musicians Without Borders course in the Netherlands. It was a short-term experience of leading musical activities in a project for orphaned children rescued from voodoo convents. In January 2023 the team travelled to Benin to work with a group of children and youth from the Maison Familiale La Solidarité (an orphanage in the village of Kénouhoué for children rescued from voodoo convents). At the time of the visit, there were 16 children and young people living there, approximately between the ages of 4 and 18. The music sessions consisted of different types of activities: generic games, musical games, and songs.

The children’s response to the activities was very positive from the beginning. They included the new songs, games, and exercises in their daily activities and playtime. The children also taught the group of foreign musicians some games, songs, and dances that they used to play together.

One of the mornings was dedicated to a session in the primary school attended by the younger Maison Familiale members. The session was planned together with the children, who decided which activities they wanted to share with their colleagues. In the short time of the session, and thanks to the support and enthusiasm of the Maison Familiale group, it was possible to sing a melody in canon (the Congolese song Banaha) and to develop many exercises with more than 100 students. Everyone’s great attention and willingness to participate exceeded the expectations (and some fears, due to the size of the group and the possible difficulties with communication). On one of the following days, a boy from the school recognized the facilitators walking past on the way to the Maison Familiale. Alone, on the street, he started to sing the song Banaha.

In addition to the musical work and games that were shared every day, there were also moments of leisure: an activity in which children could draw freely, a movie session, and several shared meals.

A collection of videos was prepared at the end of the project in order to allow the group to continue to sing and play the songs they had learned after the facilitator’s departure.

### 4.4. Greece

The part of the project in Greece was a medium-term experience of facilitating music sessions in an organization working with people living in refugee camps. Due to its geographical location, Greece is an entry point into Europe and since 2025 has received a great number of people that have left their countries, it currently has more than 237,000 forcibly displaced people (UNHCR, 2024).

Serres is a small city in Northern Greece with approximately 76,000 inhabitants. In its outskirts there are two open-air refugee camps which are made of containers and surrounded with fences which can accommodate around 1400 people, although this number is frequently exceeded. Adjacent to the camps, the NGO Lifting Hands International (LHI) organized a community centre with five tents. The LHI centre was divided into Arts and Recreation Space (A&R), Child-Friendly Space (CFS), Education Space, Female-Friendly Space (FFS), library, garden, and sports fields. Daily activities include English and German classes, yoga, music, and sports, among other activities. There were weekly art therapy sessions facilitated by a specialist. The residents of the camps are responsible for facilitating some classes/sessions as well as helping with the distribution of goods and organizing activities.

The fieldwork in Greece was part of the volunteer programme run by LHI and it was developed during two months in both A&R and CFS programmes, with the goal of facilitating music sessions and activities. When the two-month fieldwork started in February 2023, most of the population in the camps (and therefore also in the centre) was Yazidi. The Yazidis are a community from the Sinjar region, on the northern border of Iraq with Syria, that speaks Kurmanji, a Kurdish dialect. The Yazidis were victims of genocide in August 2014 by ISIS, which executed thousands of men, kidnapped approximately 7000 women and children, and destroyed the region’s infrastructure.

In A&R, the work was developed with two distinct groups: teenagers and adults. There were scheduled musical activities and free moments of leisure. Each person had a different expectation of what music sessions would be like. Some wanted to learn instruments such as guitar, piano, percussion or tambur (a traditional string instrument), and others wanted to sing. In order to create shared sessions suited for every participant, there were conversations about musical preferences and what the participants would like to do in the time spent together. The famous Italian song “Bella Ciao” was very appreciated between the groups and allowed the musical work to start and develop. The initial idea of creating a choir was not viable due to the time constraints, the impossibility of performing continuous work, and the heterogeneity of the group. Yet, it was through singing that it was possible to develop the sessions, using some musical instruments and other resources such as body percussion and rhythmical games, especially with the teenagers group.

With the adults, the sessions were naturally transformed into jam sessions of songs known by the Yazidi community, providing a place where it was possible to express a shared identity. Inspired by ideas and strategies from community music, the once-choir-conductor adapted herself to the new role of facilitator, someone that fosters hospitality, organizes the space, instruments, and all the logistics necessary for gathering people in an inviting, safe, and comfortable environment for everyone while leading the session in a shared way (the songs were chosen by the participants).

At CFS, with the children, the goal was to connect other activities (handcraft, story time, and games) with music. In addition to building instruments, playing with them, and creating musical games, songs (mainly without text or with meaningless text) were used at different times to start or end sessions, to capture children’s attention, or to establish routines.

## 5. Discussion

This journey allowed the observation and practice of collective singing in a range of contexts, with Nagham (Lebanon) and Xiquitsi (Mozambique) closer to the type of choral practice from which our experience and training emerge. The experiences in Benin and Greece strongly differ from these and indeed from what had been anticipated, as there was no choral practice, in the most commonly accepted sense, due to time and context specificities. Nevertheless, voice played a preponderant role and collective singing emerged as a strong reality. The previous choral training provided a foundation from which other approaches emerged. More important than organizing these experiences into discrete types, certainly biassed by our own European choir tradition background, it is interesting to understand how the journey contributed to enlarging our vision of what is and can be collective singing.

The entire journey was inspired by the idea of care: different contexts, strategies, repertoire, and people led to different processes and results. Music can be an escape, a way of experiencing moments of well-being in a safe space. Learning the “art of care” implies an awareness of many aspects of a large constellation: the need for flexibility and adaptation, the attitude of invitation and hospitality towards the participants, the importance of the relationships, the different ways of communication and the dialogue as a tool to achieve shared goals, the respect for cultural diversity, the presence of quality in the process and not only in the product, the existence of different types of leadership, the need of dedication, the will of creating continuity, and the implications of being an outsider. This last aspect was particularly relevant as it was necessary to respect cultural diversity and to listen, to observe, and to understand what the participants were expecting, not imposing contents and approaches. These aspects have a strong resonance from authors working in the field of community music, such as Bartleet (2023).

Different goals of the projects demanded different approaches. If in Lebanon and in Mozambique there were often moments of public performance, it became necessary to ensure that quality was present in both the process (the preparation of the concerts) and the product (the concerts). In Benin and Greece, however, all that existed was the process; there were no public presentations, and the goal was the creation of safe spaces and shared valuable moments. The approach used in community music suggested by Henley and Higgins (2020) in which the process is the excellence, and the product is the inclusion that results from this process was followed to try to ensure that all the sessions were valuable for everybody.

Adapting verbal expression to different situations to fulfil the desire and need to communicate is another interesting aspect related to care. Whereas in Mozambique this meant adapting the Portuguese for better mutual understanding, in Lebanon and Greece English was used as the main language, whereas French was the basis in Benin. It is important to mention, however, that non-verbal communication played an important role, in particular in Benin and Greece. The conjugation of sharing physical actions (meals, dancing, and games), gestures, and few words (in Arabic and Kurmanji, in the case of Greece) are aspects of a wider vocabulary that allows one to connect, express, and share. Along with the verbal communication and the musical activities, Laughter’s (2014) idea of micro-kindness was an important source of inspiration throughout the journey. We became more aware of the importance of all these small acts in our practice. They allowed us to coexist, trust and get to know each other in a deeper way, particularly in the absence of a common verbal language.

Both in Nagham Choir and in Xiquitsi, the relationships between peers and conductors/teachers are regarded as an important part of the experience. Some Nagham Choir members mentioned that they do not feel different from each other, they are like a family, and they do not understand the conflict. The importance of commitment, the relationship of respect and trust with their conductor, and the possibility of expressing their feelings through the songs they sing were some of the aspects that they cherished in the choir. At Xiquitsi, the importance of the relationships was witnessed even closer, due to the continuity of the work carried out. The rehearsals were the starting point to connect with the participants, and throughout time these bonds developed further into deep connections and friendship, nurtured by the sharing of time and space outside the context of the rehearsals. All these aspects contribute to further developing the roles normally assigned to the strict musical intervention (teacher, conductor, and facilitator) and are part of creating hospitality. Even if the relationship between facilitators and participants is one of inequality (due to the responsibility that the facilitator has in the process of welcoming), “the face-to-face encounter emerges as a friendship, an open, committed, and respectful relationship” (Higgins, 2012, p. 166).

In all these projects there was a concern to create continuity after the experience ceased, by transferring knowledge and materials to others who could continue to lead sessions, or through online communication, sharing videos and audio files, or even searching for financial and logistical support. In Xiquitsi, caring meant continuing in contact with the students even from a distance, nurturing the process initiated in Mozambique and being involved in their well-being and evolution. In Greece, however, caring meant the opposite: the LHI rules did not allow us to keep contact with people from the field as this would be likely to induce further problems in a very fragile context.

Caring meant, therefore, adapting to different circumstances, recognizing the role in each context, taking pleasure in meeting people, sharing time with them, considering the background and differences they may have, listening to them, showing curiosity, sharing knowledge, and exploring different ways of collective singing. As Bartleet (2016) states, in these settings, the most powerful and important part of the learning process comes from the intersection between relationship building and shared music making. Meeting all these amazing people and having the great opportunity and honour to make music with them taught us how to “put care at the very centre of life”, as we were challenged by the Care Manifesto (The Care Collective, 2020, p. 5).

## 6. Conclusions

This journey allowed us to experience how collective singing can be shaped in different ways. The skills acquired previously in conducting training and traditional choir work were essential throughout the journey, but it was necessary to rethink approaches and blur some boundaries between choral practice, pedagogy, and community music. The variety of experiences showed that a choir is one of the many ways of collective singing. It does not depreciate the value of choral singing. On the contrary, it helps to better understand its essence and, hopefully, to open a range of possibilities for future projects. All the interactions and experiences have led us to rethink ways of working, in a constant attempt to improve practices and actions. We believe that if there is constant dialogue and sharing, we can discuss the issues that arise together and create what is necessary (care, respect, trust, love, humanity, hospitality, and strong bonds) to make music together, with quality and meaning for everyone. In that sense, the concept of collective singing communities would be more flexible and open to different ways of singing in groups that are culturally broad, less hierarchized, and more careful with the individual that is part of the group than the term choir.

The concern for individual and group well-being was a common element, bridging the different projects within the framework of ethics of care. Care can be a reciprocal attitude that spreads and we share the vision of a society that has universal care as its ideal (The Care Collective, 2020). Collective singing communities can be one of the various care circles that can exist in society. Thus, not only within them can an ethics of care be exercised, but the same can happen from within the group to the outside, to the different communities in which its members interact. A care that goes beyond identities, ethnicities, nationalities, and borders.

We are experiencing the biggest refugee crisis since the Second World War. At the same time, borders are becoming more impenetrable, with a global increase in populist movements that disseminate racist, xenophobic, and discriminatory behaviours. Having good living and working conditions seems to be a luxury only available to some, and genocides continue to happen with the entire world watching and feeding them. It is fundamental to question our practices, whether in a rehearsal space, facilitating a session, teaching a class, within the family, in a group of friends, on social media, in what we write, on public transport, or on the streets:

*‘We’re creating a microcosm in the present of the sort of society that we hope to be more generally present, just in the way that we relate to each other: the way that we work together, the way we sing together.’*

(Campaign Choirs Writing Collective, 2018, p. 242)

The choir—as one of the shapes that collective singing can acquire—has often been referred to as an example of society organization. Other than because of its hierarchical relationships or the instrumentalization of individuality for the benefit of the group, we suggest that we transform collective singing communities into a microcosm of a society that spreads care and well-being. A care present in all spheres of our lives, privately and publicly, with acquaintances and strangers, both in the process and in the product.

## Data Availability

The original contributions presented in this study are included in the article. Further inquiries can be directed to the corresponding author.

## Notes

1 Collective singing communities is an idea that arises from Wenger-Trayner and Wenger-Trayner’s (2015) concept of communities of practice. According to the authors, what makes a community be considered a community of practice is the simultaneous presence of a domain of interest, a community, and a practice: “Communities of practice are groups of people who share a concern or a passion for something they do and learn how to do it better as they interact regularly.” (p. 2)

2 The idea of evocative auto-ethnography as purpose by Bochner and Ellis (2016) is particularly adequated to describe the narrative nature of our work.

3 Some of those virtual choirs can be accessed through these links: *Abendlied* (J. Rheinberger) https://www.youtube.com/watch?v=b-O-BDwyRE8m; *Hosi katekisa* (South African traditional hymn, with lyrics in Ronga) https://www.youtube.com/watch?v=A-qdwzeIjFE; *Shenandoah* (traditional north American, arr. J. Erb) https://www.youtube.com/watch?v=d6IVW_EIevI&t=96s; *Candombe de San Baltazar* (A. Montenegro-Yabor, arr. L. Cangiano) https://www.youtube.com/watch?v=IV26DwTvbfA; *Tristeza* (H. Lobo and Niltinho, arr. for choir W. Belan, instrumental arr. composed for this version L. Alemán and H. Tandane Jr.) https://www.youtube.com/watch?v=CC-6RMGWwZw.

4 The first of the two presentations were completely dedicated to the choirs (children, youth, and chamber). The students chose among the studied repertoire which songs they wanted to perform and in which order. The work with the children’ choir also involved creating with the students some instrumental parts, choreographies, solos and adding some harmonies to unison songs. The Chamber Choir sang: *Locus Iste* (A. Bruckner), *Crucifixus* (A. Lotti), *A soul is like a bird* (E. F. Chissano), *Sililiza* (J. Papoulis) and *Modimo* (South African song with arrangement by M. Barrett). Children choir sang: *Banaha* (Congolese song), *Sesere eyee* (traticional song from Torres Strait Islands, com edição de M. O’Leary), *Tutira Mai* (Maori song with arrangement by A. Ritchie), *Nesta rua* (Brazilian song), *Bonse aba* (traditional song from Zambia, arrangement by Cajita de Música choir) and *Ukuthula* (South African song). The concert ended with the Youth choir: *Shenandoah* (North American song with arrangement by J. Erb), *Adeus ó Serra da Lapa* (Portuguese song with arrangement by A. Teixeira), *O fortuna* (from C. Orff *Carmina Burana*) and *I sing you sing* (A. Edenrot). At the end of the concert all students sang together the South African song *Shosholoza*. The second concert was dedicated to the students that had solo pieces and also to the students that were having conducting lessons. In this situation they chose the repertoire, the staging, and everything that was related with the public presentations. Below is the programme of this concert, in the order in which it was presented: *In dulci jubilo* (christmas *carol* with arrangement by R. L. Pearsall), *Amarilli mia bella* (G. Caccini), *Intorno all’idol mio* (M. A. Cesti), *Hosanna* (Soweto Gospel Choir), *Qui sedes ad dexteram patris* (A. Vivaldi), *Non so più cosa son, cosa faccio* (W. A. Mozart), *Ave Maria* (N. Salomé), *Giunsie alfin il momento… deh, vieni* (W. A. Mozart), *Soave sia il vento* (W. A. Mozart), *Drop, drop slow tears* (O. Gibbons), *In uomini in soldati* (W. A. Mozart), *Ombra mai fu* (G. F. Händel), *Berimbau* (V. de Moraes and B. Powell with arrangement by A. Teixeira), *Ave regina* (C. Ferrari), *Stizzoso mio stizzoso* (G. B. Pergolesi), *If ye love me* (T. Tallis), *Domine Deus* (A. Vivaldi), *Vergin tutto amor* (F. Durante) and *Oh happy day* (E. R. Hawkins).
